# The Val66 and Met66 Alleles-Specific Expression of BDNF in Human Muscle and Their Metabolic Responsivity

**DOI:** 10.3389/fnmol.2021.638176

**Published:** 2021-05-05

**Authors:** Gilmara Gomes de Assis, Jay R. Hoffman, Jacek Bojakowski, Eugenia Murawska-Ciałowicz, Paweł Cięszczyk, Eugene V. Gasanov

**Affiliations:** ^1^Department of Molecular Biology, Gdansk University of Physical Education and Sport, Gdańsk, Poland; ^2^Mossakowski Medical Research Centre, Polish Academy of Sciences, Warsaw, Poland; ^3^Department of Physical Therapy, Ariel University, Ariel, Israel; ^4^Department of Neurology, Medical University of Warsaw, Warsaw, Poland; ^5^Department of Physiology and Biochemistry, Faculty of Physical Education and Sport, University School of Physical Education in Wrocław, Wrocław, Poland; ^6^Laboratory of Neurodegeneration, International Institute of Molecular and Cell Biology in Warsaw, Warsaw, Poland

**Keywords:** Val66Met polymorphism, BDNF, allele-specific gene expression, mRNA level, metabolism, VO_2_max test

## Abstract

Brain-derived neurotrophic factor (BDNF) plays an essential role in nervous system formation and functioning, including metabolism. Present only in humans, the “Val66Met” polymorphism of the BDNF gene (*BDNF*) is suggested to have a negative influence on the etiology of neurological diseases. However, this polymorphism has only been addressed, at the molecular level, in nonhuman models. Knowledge about Val66- and Met66-variant differences, to date, has been achieved at the protein level using either cell culture or animal models. Thus, the purpose of our study was to analyze the impact of the Val66Met polymorphism on *BDNF* expression in healthy humans and compare the allele-specific responses to metabolic stress. Muscle biopsies from 13 male recreational athletes (34 ± 9 years, 1.80 ± 0.08 m, 76.4 ± 10.5 kg) were obtained before and immediately following a VO_2_max test. Allele-specific BDNF mRNA concentrations were quantified by droplet digital PCR (ddPCR) in heterozygous and homozygous subjects. The results indicated that *BDNF* expression levels were influenced by the genotype according to the presence of the polymorphism. *BDNF* expression from the Met66-coding alleles, in heterozygotes, was 1.3-fold lower than that from the Val66-coding alleles. Total BDNF mRNA levels in these heterozygotes remained below the whole sample’s mean. A partial dominance was detected for the Val66-coding variant on the Met66-coding’s. *BDNF* expression levels decreased by an average of 1.8-fold following the VO_2_max test, independent of the individual’s genotype. The results of this study indicate that metabolic stress downregulates *BDNF* expression but not plasma BDNF concentrations. No correlation between expression level and plasma BDNF concentrations was found.

## Introduction

Brain-derived neurotrophic factor (BDNF) is a neurotrophin, a small secretory dimeric protein that appears in all vertebrates with a highly conserved structure (Rafieva and Gasanov, 2016). Showing the most pervasive and abundant expression throughout the brain among the neurotrophins, BDNF plays a pivotal role in cell proliferation and differentiation, and supports neural tissue formation, muscle–neuron interaction, and the organization of the central nervous system (CNS) (Friedman, 2010). In addition, BDNF is essential for neuronal outgrowth and continuous regulation of processes such as synaptic transmission, long-term potentiation, and depreciation (Lamb et al., 2015; Sasi et al., 2017). BDNF is largely expressed in neurons and neural tissue and is also produced by muscle (Matthews et al., 2009), although the role of muscle production has yet to be elucidated in humans to date.

In humans, a single nucleotide polymorphism resulting in a substitution of an adenine (A) base by guanine (G) in the BDNF gene (*BDNF*) has been identified in ∼20% of the population (Maisonpierre et al., 1991; Shimizu et al., 2004). This polymorphism is named “Val66Met” due to a Valine to Methionine substitution in the 66th amino-acid position of the synthesized protein (BDNF) with respect to the A or G genotype (Rafieva and Gasanov, 2016; Hunt et al., 2018). Considering the pivotal function of BDNF, it has been suggested that the *BDNF* Val66Met may have a role in the etiology of several neurological diseases (Egan et al., 2003; Notaras and van den Buuse, 2018; Shen et al., 2018) and psychiatric disorders (Kishi et al., 2018).

The BDNF protein (mature form, referred from here on as BDNF) is synthesized by cells as a precursor, pro-BDNF, that undergoes a multistage processing, which includes the loss of the pro-domain part containing the polymorphic 66th amino-acid position. Therefore, the Val66Met variance does not appear in the structure of released BDNF. However, a change in the pro-domain part of pro-BDNF, the Val66Met substitution, could influence the secretory pathway direction and release of BDNF (Chen et al., 2004) affecting the efficiency of BDNF synthesis and function (Bath and Lee, 2006; Pruunsild et al., 2007).

In humans, the influence of the Val66Met polymorphism has been observed through the association between Met66 genotypes and pathological changes in the morphology of specific areas of the brain (Egan et al., 2003). Importantly, the polymorphism fails to parcel out as a determinant for the development of neuropathological conditions among other factors such as ethnicity, gender, and other influences (Shimizu et al., 2004; Kishi et al., 2018; Shen et al., 2018). When examining various clinical subpopulations, there are no reliable associations between the genotype and plasma BDNF concentrations (Yoshimura et al., 2011), suggesting that the association of this polymorphism with risk for disease, based on circulating BDNF concentrations, is limited (Shen et al., 2018). Nevertheless, there appears to be a pattern in the acute stress response of individuals carrying the Met66 allele in *BDNF* that results in an upregulation in their cortisol response to stress, which may provide support for the influence of the Val66Met polymorphism in the development of stress-related disorders including neurological disease (Kishi et al., 2018; de Assis and Gasanov, 2019).

The present understanding of Val66- and Met66-variant differences, to date, has been achieved at the protein level using either cell culture or animal models (Egan et al., 2003; Chen et al., 2004; Lou et al., 2005; Anastasia et al., 2013; Uegaki et al., 2017). However, BDNF production could also be affected at the level of gene function. Serving as a template for the pro-BDNF translation, mRNA initiates BDNF production, reflecting gene activity, and is the first level of BDNF regulation. In regard to gene expression, Val66- and Met66-coding alleles may be transcribed either equally or not at all, being in competition for the mRNA synthesis machinery and potentially interfering with their expression levels. However, to the best of our knowledge, the influence of the Val66Met polymorphism on *BDNF* functioning (i.e., transcription) has never been previously analyzed.

In consideration of previous observations of BDNF responsivity to aerobic exercise (Dinoff et al., 2016; de Assis et al., 2018), we have developed a method for analyzing the gene expression of human *BDNF* regarding the Val66Met polymorphism *in vivo* using a maximal endurance exercise protocol. In order to reveal the relation between the Met66 and Val66 alleles of *BDNF*, and its impact on *BDNF* functioning (i.e., transcription), we have analyzed the quantities of BDNF mRNA in the muscle of healthy subjects at rest and following a maximal aerobic capacity (VO_2_max) test.

## Materials and Methods

In accordance with the European Union regulation 2016/679 of 27 April 2016 and the Ethical Committee of the Central Clinic Hospital of the MSWiA in Warsaw, Poland, 25 physically active healthy volunteers (37.5 ± 9.9 years, among them five women) following an explanation of all procedures, risks, and benefits, provided his or her informed consent to participate in this study. All participants were genotyped for the *BDNF* Val66Met polymorphism. Thirteen of the participants, all males (age: 34.5 ± 9 years, BMI: 22.85 ± 1.6, and VO_2_max: 54.3 ± 6.2 ml/kg/min), were included in the experiment (Table 1).

**TABLE 1:** Subject characteristics and plasma BDNF levels.

ID (Age 34 ± 9)	BMI (22.85 ± 1.6 kg/m^2^)	VO_2_max (54.33 ± 6.2 ml/kg/min)	Genotype	Plasma BDNF-pre (pg/ml)	Plasma BDNF-post (pg/ml)	Δ BDNF (pg/ml)
1	21.97	60.00	Val/Val	9300.08	7547.9	−1752.18
2	22.35	62.00	Val/Val	8220.33	7848.24	−372.09
3	21.97	59.38	Val/Met	8522.45	7214.06	−1308.39
4	26.31	41.40	Val/Val	8088.49	7456.73	−631.76
5	23.10	45.62	Val/Met	10103.24	8898.5	−1204.74
6	22.74	51.38	Val/Val	6961.47	8314.07	1352.6
7	23.70	56.06	Val/Val	8558.53	6998.88	−1559.65
8	23.89	61.40	Val/Val	7276.23	8690.76	1414.53
9	20.67	54.24	Val/Val	7623.1	8630.74	1007.64
10	22.32	57.3	Val/Val	8511.54	8831.91	320.37
11	23.39	51.46	Met/Met	9803.92	8328.3	−1475.62
12	20.23	56.38	Val/Met	7779.02	8160.82	381.8
13	24.42	49.64	Val/Val	10351.91	9005.23	−1346.68

*Val, Val66-coding; Met, Met66-coding *BDNF* allele; BMI, body mass index; VO_2_max, the maximum rate of oxygen consumption; means are given at the top of the table. BDNF-pre, plasma BDNF concentrations at rest; BDNF-post, plasma BDNF concentrations after the VO_2_max test; ΔBDNF, the BDNF-pre and BDNF-post difference.*

### Genotyping

**FIGURE 1:**
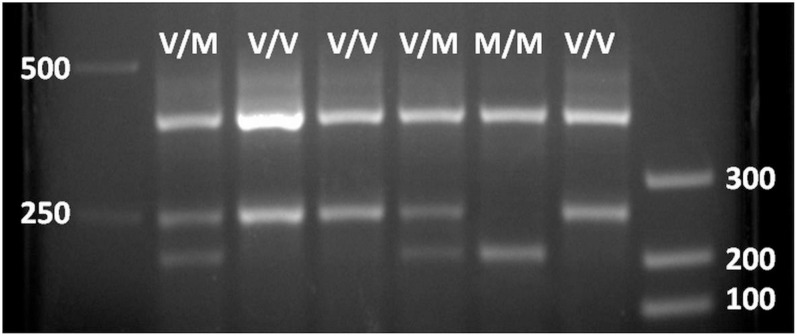
Agarose gel electrophoresis of PCR *BDNF* polymorphism analysis. 400 bp, BDNF gene full-length fragment (internal positive PCR control); 250 bp, Val66-coding BDNF gene fragment; 200 bp, Met66-coding BDNF gene fragment.

All study participants reported to the Human Performance Laboratory in the Mossakowski Medical Research Centre, Polish Academy of Science, Warsaw, Poland. During the visit, all participants were provided with a brief medical exam that included anthropometric measurements. A blood draw (3–4 ml) was obtained by a nurse from a cubital vein, divided to aliquots, and immediately embedded in dry ice and stored at −80°C until analysis. A 0.5 ml aliquot from a blood sample was used for genomic DNA (gDNA) extraction by a NucleoSpin Tissue kit (Macherey-Nagel GmbH & Co., KG, Germany) according to the manufacturer’s procedures. The resulting DNA concentrations were determined by a NanoDrop^TM^ 2000 Spectrophotometer (Thermo Fisher Scientific, United States). The gDNA was used as a template in a one-step amplified refractory mutation system PCR according to Sheikh et al. (2010). In this protocol, two primers (P1 and P2) surrounding the *BDNF’s* area of interest (polymorphism bearing ∼400 bp) and two primers (P3 and P4) corresponding to Val- and Met-coding sequences, respectively (internal to the first pair and in opposite orientation to each other) were added together with a PCR Mix Plus mixture (A&A Biotechnology, Poland) according to the manufacturer’s procedures. The reaction was performed in a C1000 Touch Thermal Cycler (Bio-Rad, United States). PCR results were analyzed in a 6% polyacrylamide or 2.5% agarose gel-electrophoresis with ethidium bromide staining. Images were generated by Bio-Rad Gel Documentation System. Results showed the full-length *BDNF*-specific product (∼400 bp) and the corresponding products to the presence of Val (∼250 bp) and Met (∼200 bp) in *BDNF* variants (see Figure 1).

### Exercise Protocol

Participants reported to the laboratory and rested comfortably in a supine position for 30 min. Heart rate and blood pressure were obtained prior to the initial muscle biopsy. Following the biopsy (∼15 min), participants performed a maximum aerobic capacity (VO_2_max) test using the Bruce treadmill protocol (Bruce et al., 2004). Change in both the gradient and the speed of the treadmill occurred every 3 min (seven stages) that began at 2.7 kph/10% gradient and progressed up to 8.9 kph/20% grade, or until the participants’ volitional exhaustion. Immediately (30–60 s) following the conclusion of the VO_2_max test, a second muscle biopsy was obtained. Blood samples were collected from an antecubital vein at the same time-point of the muscle biopsies.

### Muscle Biopsy Procedure

Approximately 50 mg (wet weight) of skeletal muscle from *Vastus lateralis* was obtained per sampling by suction using a semiautomatic needle biopsy (14 g, 100 mm). The sample was immediately embedded in dry ice and stored at −80°C until analysis. All procedures occurred between 3 p.m. and 5 p.m. (GMT+2).

### RNA Extraction

Muscle samples were manually homogenized in Eppendorf tubes and mixed with 0.5 ml of a TRIzol reagent (T9424, Sigma-Aldrich). The TRIzol-chloroform RNA extraction was performed according to the manufacturer’s protocol, and the total RNA was dissolved in 0.03 ml of RNAse-free water; RNA concentrations were determined by a NanoDrop^TM^ 2000 Spectrophotometer, and the RNA solutions were stored at −80°C. One microgram of RNA was later used for cDNA synthesis in 0.02 ml of the reaction mixture using the iScript^TM^ Reverse Transcription kit (Bio-Rad) according to the manufacturer’s protocol. Subsequently, 1 μl of the cDNA solution was used as a template for ddPCR analyses.

### Droplet Digital PCR (ddPCR) Analysis

A housekeeping gene was primarily established as a reference for the patterns of *BDNF* expression in the ddPCR^TM^ system (de Assis et al., 2020). For this, the cDNA of three subjects’ samples (one woman) both at rest and post VO_2_max were applied in 20 μl reactions for the Bio-Rad PrimePCR Pathway Plate—Reference Genes H96, Human—containing 14 housekeeping gene candidates recommended for muscle tissue analysis, internal PCR, reverse transcription, RNA quality, and gDNA admixture controls. RT-PCRs were performed in triplicate according to the manufacturer’s protocol (Bio-Rad, United States). The beta-2-microglobulin (*B2M*) and the ribosomal protein S18 (*RPS18*) were among the three of the most reliable candidate genes applicable for ddPCR^TM^ analysis [inter- and intraindividual threshold cycle, C(t), difference did not exceed 2.5 cycles]. The 13 cDNA samples were then applied to the ddPCR^TM^ system with an allele-specific BDNF assay containing hydrolyzed probes (TaqMan) with both HEX (hexachlorofluorescein) and FAM (6-carboxyfluorescein) detection of Val- and Met-coding alleles, respectively (ddPCR^TM^ Mutation Assay: rs6265, dHsaMDS320493890), using PrimePCR^TM^ ddPCR^TM^ Supermix for Probes (no dUTP), as well as the PrimePCR^TM^ ddPCR^TM^ Expression Probe assay, *B2M*, Human (HEX-probe detection, qHsaCPE5053101) in parallel (all designed by Bio-Rad). RNA samples without a reverse transcription step were also used in parallel as a template, in the same quantity according to the RNA template in cDNA samples as a genomic DNA (gDNA) admixture control. The quantities of reaction products in these samples were subtracted from the corresponding samples analyzed for *BDNF* expression (de Assis et al., 2020). The gDNA of Val66Met heterozygotes was used as a control of the *BDNF* allele ratio and as a positive control of the reaction. The ratios between Val and Met-alleles of *BDNF* in gDNA samples were 1 ± 0.03.

To exclude the reference gene influence, the second part of mRNA samples were treated by DNase I (M0303, NEB, United States) according to the manufacturer’s protocol and used for cDNA synthesis as described above. One microliter of cDNA solution was used in ddPCR for the allele-specific *BDNF* expression analysis with the RPS18 gene as a reference using the PrimePCR^TM^ Probe assay, *RPS18*, Human (FAM-detection, qHsaCEP0040177, Bio-Rad). *B2M* and *RPS18* expression was also compared in the same reaction using FAM- and HEX-fluorescence detection. All reactions were performed in duplicate.

### Plasma BDNF Analysis

To measure the BDNF concentration in plasma, blood samples were collected and drawn into tubes containing ethylene-diaminetetraacetic acid (EDTA) and stored at 4°C for 2–3 h. Then samples were centrifuged at 2,500 × *g* for 15 min at 4°C, and the supernatants were stored at −80°C until analysis. During analysis, thawed samples were centrifuged in Eppendorf tubes at 10,000 × *g* for 10 min. BDNF concentrations in plasma samples were analyzed based on the manufacturer’s guidance by the sandwich enzyme-linked immunosorbent assay (ELISA) Human BDNF PicoKine kit (Boster Bio, United States). The absorbance at 450 nm was measured with a Spark multimode microplate reader (Tecan, Switzerland) to determine BDNF values using the lyophilized human BDNF (Boster Bio, United States) dilutions as a standard. The ELISA kit sensitivity was <15 pg/ml, the coefficient of variation was <7.5%, and all blood samples were analyzed in duplicate.

### Statistical Analysis

Pearson product-moment correlations were applied to the pre-post VO_2_max values of BDNF/B2M^∗^10^3^ expression and BDNF plasma concentrations, as well as for their concentration deltas. The association of age, BMI, and VO_2_max with BDNF expression and plasma BDNF was also verified by Pearson correlation analysis. Paired Student’s *t*-tests were used to verify pre-post differences in BDNF expression and BDNF blood concentrations. Two-way analysis of variance (ANOVA) was applied to verify for significance in Val66Met genotype influence on pre-post VO_2_max *BDNF* expression changes and allele-specific expression difference in heterozygotes. In the event of a significant *F* ratio, Bonferroni *post hoc* analysis was used to examine pairwise differences.

To compare the allele-specific expression in the heterozygotes regardless of conditions, the means of single allele mRNA levels were calculated in both pre- and post-VO_2_max test (pre- and post-means for each allele). Next, the levels of Val66- and Met66-coding alleles’ expression in pre- and post-VO_2_max conditions were standardized by calculating their ratios with their pre- and post-means, respectively. The standardized numbers of Val66- and Met66-coding alleles’ expression were then analyzed by a paired *t*-test. An alpha level of *p* ≤ 0.05 was used to determine statistical significance. All the data are presented as mean ± SD.

## Results

Characteristics and genotypes of the study participants including plasma BDNF concentrations are presented in Table 1. Comparison between *B2M* and *RPS18* showed that these genes’ expression patterns were fully coherent, with no difference in pre-post VO_2_max test results (Supplementary Figures 1, 2). Therefore, all the calculations of *BDNF* expression quantities were related to B2M mRNA quantities. The results of BDNF mRNA/B2M^∗^10^3^ expression analyses from skeletal muscle samples showed a high homogeneity at rest (1.78 ± 0.53 BDNF/10^3^ B2M mRNA molecules) and even higher homogeneity after the VO_2_max test conditions (0.98 ± 0.33 BDNF/10^3^ B2M mRNA molecules) (Table 2). The BDNF mRNA concentrations ranged from 0.528 to 2.982 molecules of BDNF per 10^3^ molecules of B2M. Neither the individual differences nor the changes evoked by the VO_2_max test exceeded a threefold change in BDNF mRNA levels.

**TABLE 2:** Allele-specific quantification of BDNF mRNA in human muscle tissue by ddPCR with B2M mRNA as a reference.

ID	Genotype	Allele-specific BDNF mRNA*				B2M/10 mRNA*		*BDNF mRNA/1000 B2M mRNA*			
			
Condition ->		Pre-Val	Pre-Met	Post-Val	Post-Met	Pre	Post	Pre-Val	Pre-Met	Post-Val	Post-Met
1	Val/Val	1.09	0	3.27	0	83.8	444	1.305	0	0.736	0
2	Val/Val	5.48	0	2.95	0	279	235	1.962	0	1.255	0
3	Val/Met	1.85	1.17	2.08	1.43	220	406	0.840	0.530	0.512	0.346
4	Val/Val	0.62	0	2.64	0	49.6	500	1.253	0	0.528	0
5	Val/Met	1.55	1.11	1.35	1.2	170	268	0.913	0.655	0.503	0.376
6	Val/Val	7.02	0	2.59	0	367	368	1.912	0	0.704	0
7	Val/Val	2.64	0	1.40	0	155	163	1.701	0	0.860	0
8	Val/Val	3.04	0	6.43	0	101.9	435	2.982	0	1.478	0
9	Val/Val	5.17	0	8.47	0	245	714	2.109	0	1.186	0
10	Val/Val	2.70	0	1.80	0	224	302	1.205	0	0.596	0
11	Met/Met	0	1.53	0	1.97	60.8	130	0	2.522	0	1.513
12	Val/Met	0.90	0.63	6.33	5.03	115	1,201	0.783	0.551	0.527	0.419
13	Val/Val	4.40	0	5.43	0	230	428	1.913	0	1.269	0

*Expression values are disposed in (copies/μl of reaction mixture), 1 μl of reaction mixture corresponds to 0.625–1.25 mg of muscle tissue. Pre-Val, BDNF Val66-allele expression values in rest; post-Val, Val66-allele expression values after VO_2_max test; pre-Met, BDNF Met66-allele expression values in rest; post-Met, Met66-allele expression values after VO_2_max test. B2M, beta-2-microglobulin expression values, pre- and post.

**FIGURE 2:**
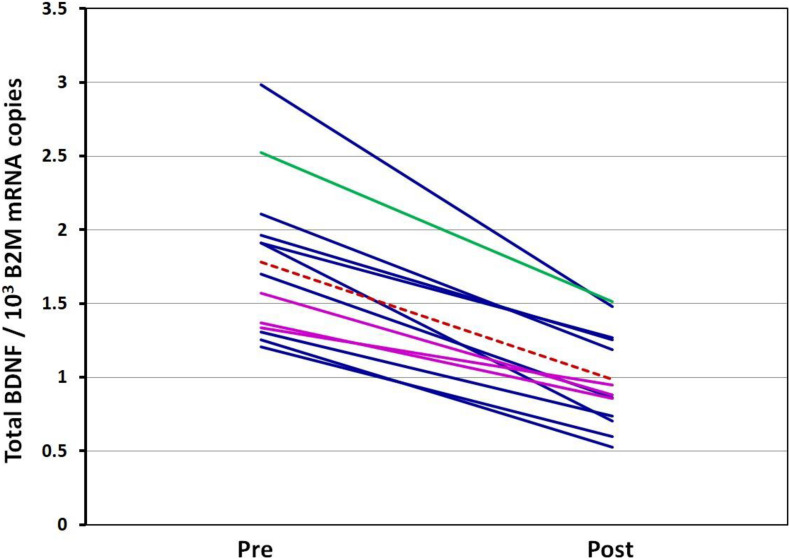
Spaghetti plot of *BDNF* expression level change. Pre—Total *BDNF* expression in rest conditions; Post—Total *BDNF* expression after VO_2_max test. Val66Val homozygotes are colored dark blue, Val66Met heterozygotes: magenta, Met66Met homozygote: green, Mean: the dotted red line. All BDNF expression values are related to B2M mRNA.

**FIGURE 3:**
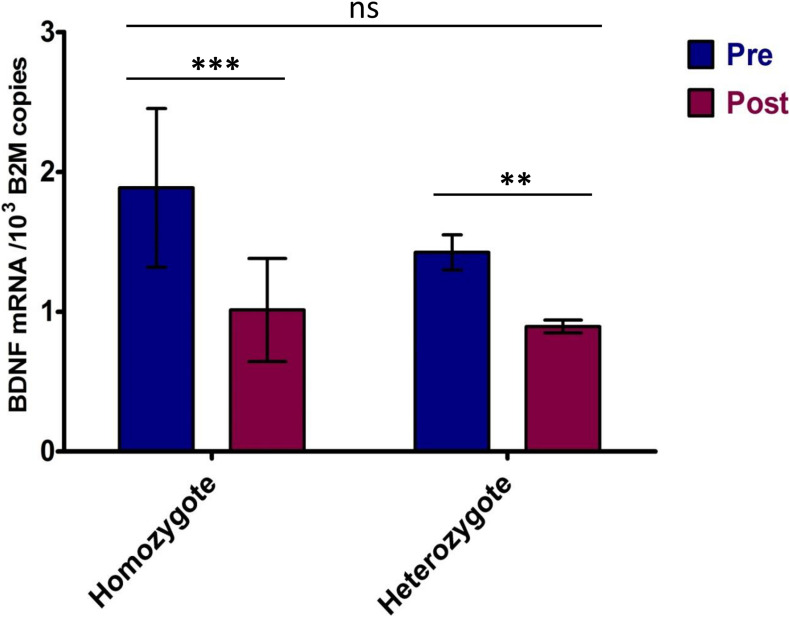
*BDNF* expression in homo- and heterozygotes in response to maximum effort test. Pre—Total *BDNF* expression in rest conditions; Post—Total *BDNF* expression after VO_2_max test. Significance levels marked by asterisks, where **p < 0.05, ***p < 0.01. Bars are given with ± SD.

The BDNF mRNA expression was significantly higher at rest compared to post-VO_2_max test conditions (*p* < 0.001). A 44 ± 9.7% decrease in BDNF mRNA levels following the VO_2_max test was observed (Table 3). There was a strong positive correlation between resting- and post-VO_2_max test values of BDNF mRNA (*r* = 0.859; *p* < 0.001). Participants whose *BDNF* expression levels were above the group average at rest maintained *BDNF* expression levels above the group mean following the VO_2_max test, whereas those whose expression levels were below the mean remained below the average *BDNF* expression levels of the group. A strong negative correlation was detected between resting *BDNF* expression levels and the pre-post changes in *BDNF* expression (pre/post Δ) (*r* = −0.838; *p* < 0.001), indicating a greater fall in *BDNF* expression levels for those displaying higher basal levels of *BDNF* expression. ANOVA performed between *BDNF* Val66Met homo- and heterozygotes indicated a significant effect of the VO_2_max test on gene expression [*F*(1,22) = 12.02, *p* = 0.002, part. eta sq. = 0.33] with no influence of the genotype (*p* = 0.1656). *Post hoc* analysis confirmed the significant effect of the VO_2_max test on *BDNF* expression (*p* < 0.001) and the absence of significant genotype influence (*p* > 0.05) (Figures 2, 3).

**TABLE 3:** Pre-Post VO_2_max test differences in *BDNF* expression related to B2M mRNA.

ID	*Allele-specific BDNF expression decrease (%)*				
	
	*Val*	*Met*	*Val* Mean, SD	*Met* Mean, SD	Total mean, SD
1	−43.6		−45.5 ± 9.4%*	−35.3 ± 8.4%*	−44.5 ± 9.7%
2	−36.0				
3	−39.9	−34.7			
4	−57.9				
5	−44.9	−42.6			
6	−63.2				
7	−49.4				
8	−50.4				
9	−43.8				
10	−50.6				
11		−40.0			
12	−32.6	−23.7			
13	−33.6				

Val, Val66-coding *BDNF* allele; Met, Met66-coding *BDNF* allele. *p-value of *Val*/*Met* mean difference is 0.09.

Regarding allele specificity, Met66-coding BDNF mRNA levels in heterozygotes quantified 76.1 ± 11.2% of Val66-coding mRNA level values independent of metabolic conditions, which means 1.3-fold lower. This was confirmed by a comparison of the allele-specific expression levels standardized with the condition-specific expression means (*p* < 0.001) (Table 4).

**TABLE 4:** Val66-Met66 allele *BDNF* expression ratio in heterozygotes.

ID	*BDNF* expression, Val/Met ratio (Met, % of Val)			Normalized to pre- and post-means *BDNF* allele expression*			
		
	Pre	Post	Mean, SD	Pre-Val	Pre-Met	Post-Val	Post-Met
3	1.58 (63.3%)	1.04 (96.1%)	1.33 ± 0.18 (76.1 ± 11.2%)	1.18	0.74	1.15	0.77
5	1.39 (71.9%)	1.30 (76.9%)		1.28	0.92	1.12	0.84
12	1.42 (70.4%)	1.26 (79.4%)		1.10	0.77	1.18	0.94

Pre, post, rest, and post-VO_2_max test conditions. *p-value of normalized *Val*/*Met* mean difference is 0.0002. BDNF expression values related to B2M mRNA.

**FIGURE 4:**
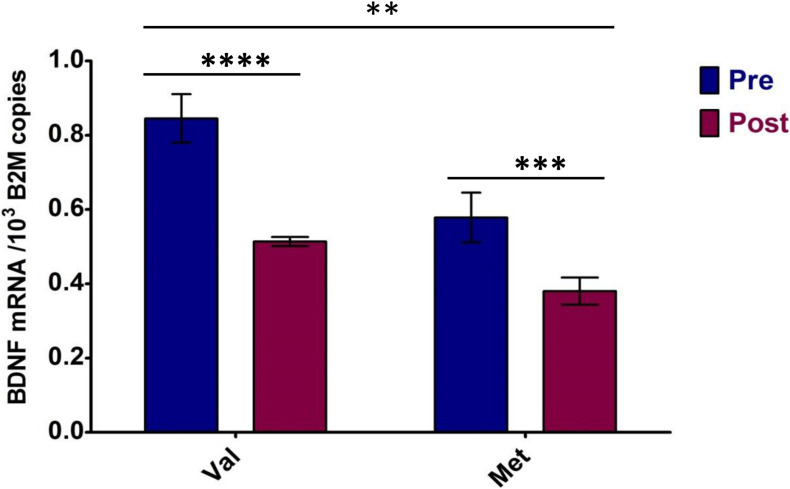
Allele-specific *BDNF* expression of heterozygotes in response to maximum effort test. Pre—Total *BDNF* expression in rest conditions; Post—Total *BDNF* expression after VO_2_max test. Val, Val66-coding BDNF mRNA; Met, Met66-coding BDNF mRNA. Significance levels marked by asterisks, where **p < 0.05, ***p < 0.01, ****p < 0.001. Bars are given with ± SD.

Two-way ANOVA performed between the Val66- and Met66-coding allele expression levels in *BDNF* heterozygotes pre- and post-VO_2_max test confirmed both the metabolic stress effect [*F*(1,8) = 82.35, *p* < 0.0001, part. eta sq. = 0.58] on *BDNF* expression and the *BDNF* allele expression difference [*F*(1,8) = 47.05, *p* = 0.0001, part. eta sq. = 0.33] (see Figure 4). In addition, a significant interaction [*F*(1,8) = 5.19, *p* = 0.052, part. eta sq. = 0.04) was noted between Val66 and Met66 allele expression differences in response to the VO_2_max test.

The pre-/post-VO_2_max test changes in *BDNF* expression, as well as the Val66-/Met66-coding allele relations, were confirmed in independent measurements with *RPS18* expression as a reference (Table 5). A 38.3 ± 12.5% decrease in BDNF mRNA levels following the VO_2_max test was observed (Supplementary Figure 2). Met66-coding BDNF mRNA levels in heterozygotes quantified 78.5 ± 9.2% of Val66-coding mRNA level values (1.29 ± 0.14-fold lower) independent of metabolic conditions (Table 5).

**TABLE 5:** Allele-specific quantification of BDNF mRNA in human muscle tissue by ddPCR with RPS18 mRNA as a reference, pre-post VO_2_ max test *BDNF* differences, and Val66-Met66 allele *BDNF* expression ratio in heterozygotes related to RSP18 mRNA.

ID	Genotype	Allele-specific BDNF mRNA*				RPS18/10 mRNA*		*BDNF mRNA/10,000 RPS18 mRNA*				*BDNF expression decrease (%)*		*BDNF expression, (Met, % of Val)*	
					
Condition ->		Pre-Val	Pre-Met	Post-Val	Post-Met	Pre	Post	Pre-Val	Pre-Met	Post-Val	Post-Met	Val	Met	Pre	Post
1	Val/Val	3.4	0	1.4	0	1,440	1,036	2.36	0	1.35	0	−42.8			
2	Val/Val	3.3	0	5	0	1,008	2,566	3.27	0	1.95	0	−40.5			
3	Val/Met	2.6	1.7	2.1	1.5	1,006	1,057	2.58	1.69	1.99	1.42	−23.1	−16.0	65.4	71.4
4	Val/Val	3.1	0	0.8	0	1,253	764	2.47	0	1.05	0	−57.7			
5	Val/Met	1.7	1.3	1.6	1.4	1,453	1,994	1.17	0.89	0.80	0.70	−31.4	−21.5	76.5	87.5
6	Val/Val	7.9	0	3.4	0	2,322	2,024	3.40	0	1.68	0	−50.6			
7	Val/Val	2.2	0	3.3	0	1,088	2,516	2.02	0	1.31	0	−35.1			
8	Val/Val	6.5	0	3.1	0	1,203	1,017	5.40	0	3.05	0	−43.6			
9	Val/Val	7.5	0	5.7	0	2,361	2,452	3.18	0	2.32	0	−26.8			
10	Val/Val	6.4	0	5	0	1,620	1,924	3.95	0	2.60	0	−34.2			
11	Met/Met	0	6.1	0	8.8	1,454	3,200	0	4.20	0	2.75	0	−34.5		
12	Val/Met	3.8	3.1	2.7	2.4	1,507	2,396	2.52	2.06	1.13	1.00	−55,3	−51.3	81.6	88.9
13	Val/Val	4.1	0	2.9	0	2,275	3,095	1.80	0	0.94	0	−48,0			
Mean												−38.3 ± 12.5		78.5 ± 9.2	

*Expression values are disposed in (copies/μl of reaction mixture), 1 μl of reaction mixture corresponds to 0.625–1.25 mg of muscle tissue. Pre-Val, BDNF Val66-allele expression values in rest; post-Val, Val66-allele expression values after VO_2_max test; pre-Met, BDNF Met66-allele expression values in rest; post-Met, Met66-allele expression values after VO_2_max test; RPS18, ribosomal protein S18 gene expression values, pre- and post.

**FIGURE 5:**
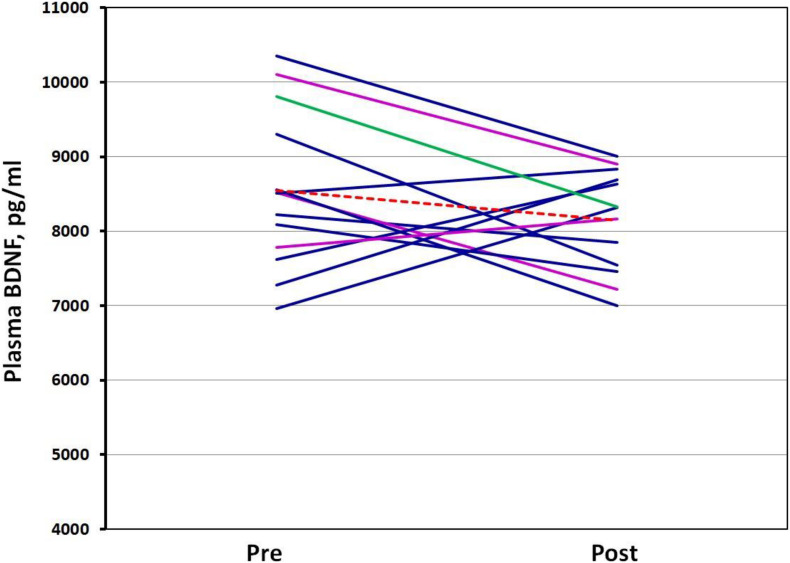
Spaghetti plot of plasma BDNF level change. Pre—plasma BDNF concentrations in rest conditions; Post—plasma BDNF concentrations after VO_2_max test. Val66Val homozygotes are colored dark blue, Val66Met heterozygotes: magenta, Met66Met homozygote: green, Mean: the dotted red line.

The differences between resting and post-VO_2_max test plasma BDNF concentrations were not significantly different. In addition, the genotype did not appear to influence plasma BDNF concentrations. However, a strong negative correlation (*r* = −0.821; *p* < 0.001) was noted between plasma BDNF concentrations pre-VO_2_max test and the changes evoked by the test—the ΔBDNF (plasma post-BDNF – pre-BDNF). This suggests that participants with higher resting plasma BDNF concentrations experienced a decrease in post-test BDNF concentrations, while those with lower plasma BDNF concentrations experienced an increase in post-exercise BDNF concentrations (Figure 5). A moderate negative correlation was noted between the *BDNF* expression level changes (pre/post Δ) and plasma BDNF ones (ΔBDNF) (*r* = −0.531, *p* = 0.062). This means that a greater decrease in the *BDNF* expression level was associated with an increase in the plasma BDNF concentrations resulting from the VO_2_max test, while a lighter decrease in the *BDNF* expression tended to reflect a decrease in plasma BDNF concentrations. No association was noted between BDNF mRNA and plasma concentrations of BDNF at rest (*r* = −0.15, *p* = 0.634) nor post-VO_2_max assessments (*r* = 0.36, *p* = 0.221), as well as between any of the participants’ physiological parameters (VO_2_max, height, weight, and age).

## Discussion

Our results indicated that BDNF expression in muscle was within a narrow range among all participants, regardless of the genotype and conditions (rest and metabolic stress), suggesting that muscle *BDNF* expression is very stable. The absolute quantification of muscle *BDNF* expression by ddPCR provided an opportunity to detect relatively small changes resulting from different metabolic conditions and genotype-induced expression variations. The effect of exercise on changes in *BDNF* expression levels was not dependent on the participants’ age or genotype suggesting a consistent response. This is supported by previous research of Matthews et al. (2009) who reported an increase in *BDNF* expression from 2 to 24 h following endurance exercise (120 min cycling at 60% of VO_2_max) using quantitative RT-PCR. However, immediately after the bout of exercise, *BDNF* expression levels were shown to decrease at the same time-point as our results. It is likely that an initial decay in *BDNF* expression is seen during an acute metabolic stress, which is followed by a compensatory rise during recovery. The results of the present study indicated that the higher the pre-exercise *BDNF* expression levels, the more pronounced their decline during exercise. This perhaps is suggestive of a stability feature for *BDNF* expression, as well as an immediate sensitivity to metabolic changes.

Plasma BDNF concentrations failed to correlate with *BDNF* expression levels at both rest and post-exercise, with no one-directional changes noted in plasma BDNF following the exercise stress. Instead, an increase in plasma BDNF concentrations was noted in the participants with lower resting BDNF concentrations in response to the VO_2_max test, while the participants with higher plasma BDNF concentrations at rest experienced a decrease following maximal effort exercise. This bidirectional response, which appears to be related to resting BDNF concentrations, may reflect a stabilizing tendency to maintain circulating BDNF concentrations within a certain biological range during metabolic stress, such as exercise. Another potential explanation is also related to the exhaustive exercise protocol in which participants have a specific switch point that is activated when the metabolic system moves from predominantly aerobic to anaerobic. This is supported by previous studies that reported a difference in the circulating BDNF response to aerobic vs. anaerobic exercise (Dinoff et al., 2016; de Assis et al., 2018).

A weak correlation was observed between changes in both expression and circulating levels of BDNF. Those participants showing greater changes in gene expression experienced smaller changes in plasma BDNF concentrations. This suggests negative regulatory feedback from circulating BDNF concentrations to its gene expression, suggesting an indirect or delayed connection between *BDNF* expression and blood concentrations. The discrepancy between *BDNF* expression and secretion may be related to possible differences in the response of BDNF producing blood and muscle cells to a metabolic stress, especially if the latter are not a source of circulating BDNF (Matthews et al., 2009). Furthermore, the appearance of BDNF in the circulation requires a considerable time lag from mRNA translation to dimeric active protein secretion. BDNF released by cells into the circulation involves secretory vesicle trafficking and a complex multistage process that includes the cleavage of pro-BDNF with the pro-domain removal. Increases in BDNF expression leads to an increase in pro-BDNF synthesis with a slow accumulation in secretory vesicles where pro-BDNF conversion to BDNF begins (Chen et al., 2004; Lou et al., 2005; Pruunsild et al., 2007; Rafieva and Gasanov, 2016). Thus, changes in *BDNF* expression levels are unlikely to immediately affect BDNF production.

The release of BDNF in response to metabolic stress and other types of signals is characteristic of the regulated pathway of neurotrophin secretion (Sasi et al., 2017). It represents a stimulus-dependent liberation by cells of secretory vesicles containing pre-synthesized BDNF that results in increases in circulating BDNF concentrations (Chen et al., 2004; Lou et al., 2005; Rafieva and Gasanov, 2016). The temporal gap between BDNF mRNA synthesis and BDNF release is dependent on the stimulus but not on the current level of *BDNF* expression. This likely contributes to the lack of an association between BDNF gene expression levels and changes in circulating concentrations.

The discrepancy between *BDNF* expression and circulating BDNF concentrations should be addressed with respect to the efficiency in the conversion of pro-BDNF to BDNF. Secretory vesicles contain unprocessed pro-BDNF; once secreted, it is subsequently cleaved to BDNF by blood and tissue proteases, increasing the gap between gene expression and mature factor appearance (Friedman, 2010; Rafieva and Gasanov, 2016). The portion of secreted unprocessed pro-BDNF may potentially increase when metabolically stimulated. This has been supported by investigators reporting an increase in the pro-BDNF/BDNF ratio during an acute aerobic stress (Brunelli et al., 2012). Thus, relying solely on blood BDNF concentrations may not provide the full spectrum of BDNF production. Measuring *BDNF* expression levels, as well as pro-BDNF circulating concentrations, may provide a more complete picture.

To the best of our knowledge, this appears to be the first investigation to demonstrate that the two alleles of human *BDNF* are expressed simultaneously in both non-polymorphic and polymorphic individuals, carrying Val66- and Met66-coding alleles. By comparing the total *BDNF* expression in all participants and detecting the allele-specific expression in the heterozygotes, we were able to conclude that both alleles of human *BDNF* are active. At the same time, our data showed an inequality between Val66 and Met66 *BDNF* allele activity. Despite the low number of heterozygotes occurring in healthy volunteers (3 out of 25 participants), the difference between the Val66-coding and the Met66-coding BDNF mRNA levels was detected in each one of the heterozygotes, at rest or during a metabolic stress (e.g., maximal exercise).

Gene activity (i.e., effectiveness of transcription) depends on the *cis-*regulatory elements represented in the gene and its neighboring DNA sequence, as well as on *trans-*regulatory elements represented by a set of regulatory genes. In the cellular environment of a heterozygous organism, all the *trans-*acting elements are shared between the two alleles of a gene so that their interaction with the gene’s alleles (in our case, *BDNF*) is determined by the relative effectiveness of the alleles’ *cis-*regulatory elements. By this, the heterogeneity in the gene alleles’ expression depends on its sequence only. Analyzing the transcription effectiveness of two different *BDNF* alleles in one subject, excluding differences in non-genetic and *trans-*acting factors, we compared the alleles’ relative activity, i.e., dominant/recessive relations (Yan and Zhou, 2004). Our results suggested that the Val66Met polymorphism is a *cis-*acting element affecting the gene expression in which the Val66-coding allele variant exhibits a partial dominance on the Met66-coding allele variant.

To exclude the metabolic stress factor, a mathematical averaging of *BDNF* expression at rest and post-exercise in heterozygotes was performed. A difference in Val66 and Met66 BDNF gene alleles was confirmed. The dominant-recessive relation of *BDNF* Val66 and Met66 BDNF alleles was also supported by the expression pattern of the Met66-homozygote subject, where in the absence of Val66-coding allele suppression, it demonstrated one of the highest levels of *BDNF* expression.

The total expression of *BDNF* in Met66-heterozygotes remained below the mean expression level of the whole sample. A trend for a stabilizing shape was also observed for total *BDNF* expression—the higher the resting expression levels, the greater the decrease following maximal exercise. Interestingly, this effect was also attributed to an allele-specific reaction. The Val66-allele, which exposed higher levels of expression in comparison to the Met66 one, demonstrated the more pronounced exercise-induced decrease, while the Met66-allele’s expression decline after exercise was milder.

There are several limitations to our study. Our observations are based on comparing three heterozygotes to 10 homozygote participants. Although results are consistent, further investigations are needed in larger samples of genotyped populations. As allele interplay is possible in heterozygotes only, this group should be analyzed apart from the homozygotes where no such *BDNF* expression suppressive effect is possible. Therefore, association studies dividing populations traditionally into groups of Val66-homozygotes and Met66-carriers (with Met66-homozygotes and Val66Met-heterozygotes inside) can be led to a diminishment of significant findings and/or incorrect interpretations. The results of our study support the hypothesis that the Val66Met polymorphism impacts the BDNF gene function (Egan et al., 2003; Chen et al., 2004; Bath and Lee, 2006; Kishi et al., 2018; Notaras and van den Buuse, 2018; Shen et al., 2018; Skibinska et al., 2018; Toh et al., 2018). However, alteration in *BDNF* expression levels does not appear to directly impact BDNF release/secretion. It appears that the post-translation processes prior to BDNF release can be differentially affected by the presence of Methionine, which might imply the Val66Met polymorphism influence on BDNF release and functions (Egan et al., 2003; Chen et al., 2004; Yoshimura et al., 2011; Uegaki et al., 2017).

## Conclusion

Our study showed that *BDNF* expresses both alleles simultaneously. Results indicated that the expression of *BDNF* immediately after maximal exercise decreases 1.8 ± 0.4-fold, regardless of the genotype. Resting *BDNF* expression levels positively correlate with post-exercise levels and negatively correlate with the magnitude of exercise-induced changes. Furthermore, *BDNF* expression levels do not correlate with plasma BDNF. The levels of *BDNF* expression appear to be modulated by the Met66 allele presence in heterozygotes. The Val66-allele variant exhibits a partial dominance over the Met66-allele variant. We believe that these findings further our understanding on the mechanisms of BDNF production and function. To elucidate the physiological implications of Val66Met polymorphism on nervous systems-related pathologies, further studies on *BDNF* expression and protein release must be addressed to specific genotyped populations.

## Data Availability Statement

The datasets generated during and/or analyzed during the current study are available in the Zenodo repository, https://zenodo.org/record/3633677#X8EAyc1KiUk.

## Ethics Statement

This study was performed in accordance with the European Union regulation 2016/679 of 27 April 2016 and the 96 Ethical Committee of the Central Clinic Hospital of the MSWiA in Warsaw, Poland.

## Author Contributions

GA, PC, and EG: conceptualization. GA, JB, and EG: sample collection and analysis, and resources. GA and EG: data curation and project administration. GA, JH, and EG: writing—original draft preparation. EM-C and PC: writing—review and editing, and funding acquisition. PC: supervision. All authors contributed to the article and approved the submitted version.

## Conflict of Interest

The authors declare that the research was conducted in the absence of any commercial or financial relationships that could be construed as a potential conflict of interest.

## Supplementary Material

Supplementary Figure 1 *B2M* and *RPS18* expression levels in samples. cDNA samples numbered; mRNA samples represent the gDNA admixture, and reaction specificity control.

Supplementary Figure 2 *B2M* and *RPS18* expression level ratio stability in Pre- and Post-VO_2_max test samples.

Supplementary Figure 3 Spaghetti plot of *BDNF* expression level change related to RPS18 mRNA. Pre—Total *BDNF* expression in rest conditions; Post—Total *BDNF* expression after VO_2_max test. Val66Val homozygotes are colored dark blue, Val66Met heterozygotes: magenta, Met66Met homozygote: green, Mean: the dotted red line.
