# Supplementary figures and images for: The Val66 and Met66 Alleles-Specific Expression of BDNF in Human Muscle and Their Metabolic Responsivity
Source: Front Mol Neurosci. 2021 May 5;14:638176. doi: 10.3389/fnmol.2021.638176 (PMC8131668; doi:10.3389/fnmol.2021.638176)

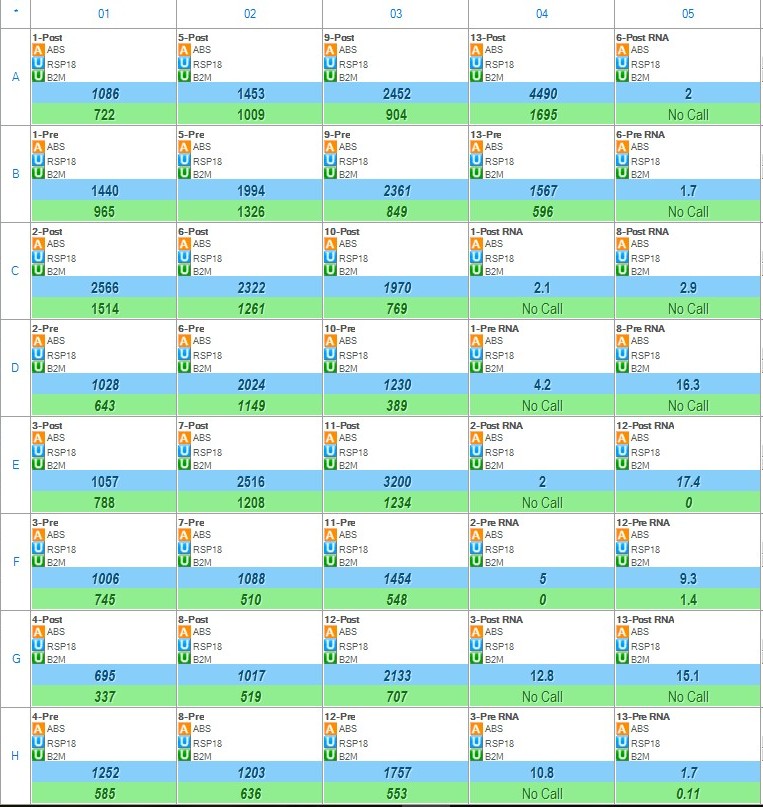

Supplement: Supplementary Figure 1 — B2M and RPS18 expression levels in samples. cDNA samples numbered; mRNA samples represent the gDNA admixture, and reaction specificity control. [file Image_1.jpg]

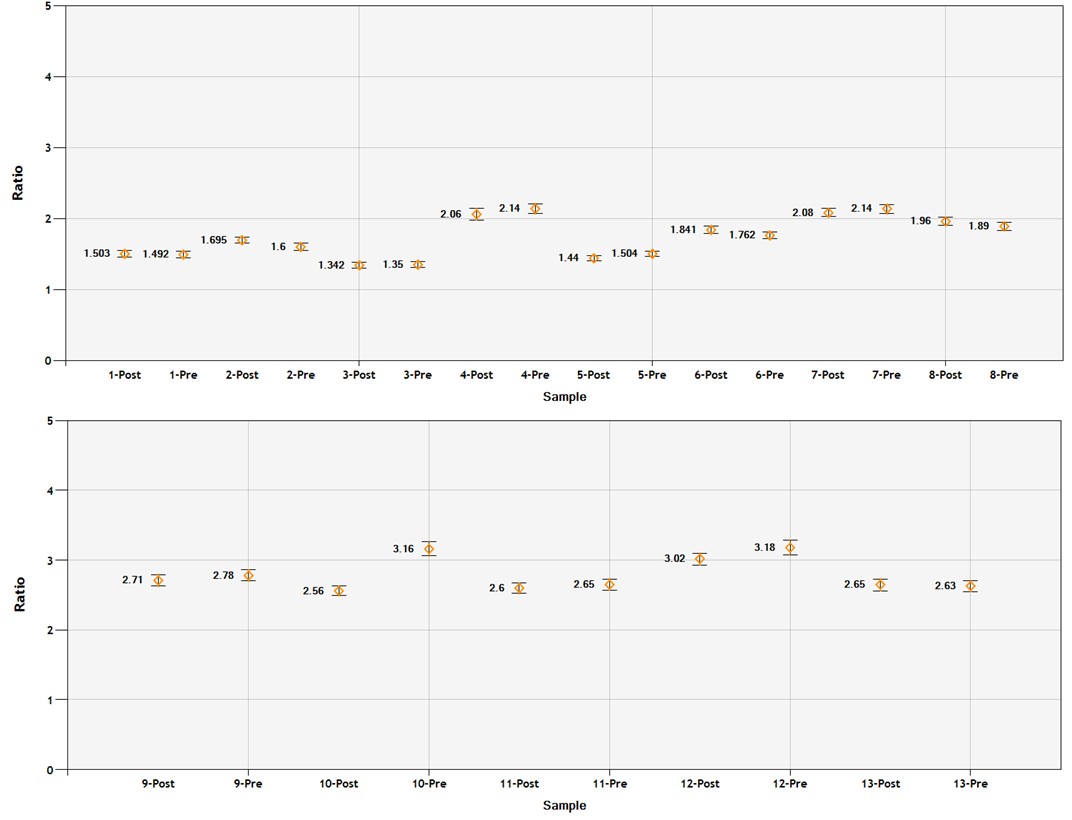

Supplement: Supplementary Figure 2 — B2M and RPS18 expression level ratio stability in Pre- and Post-VO2max test samples. [file Image_2.jpg]

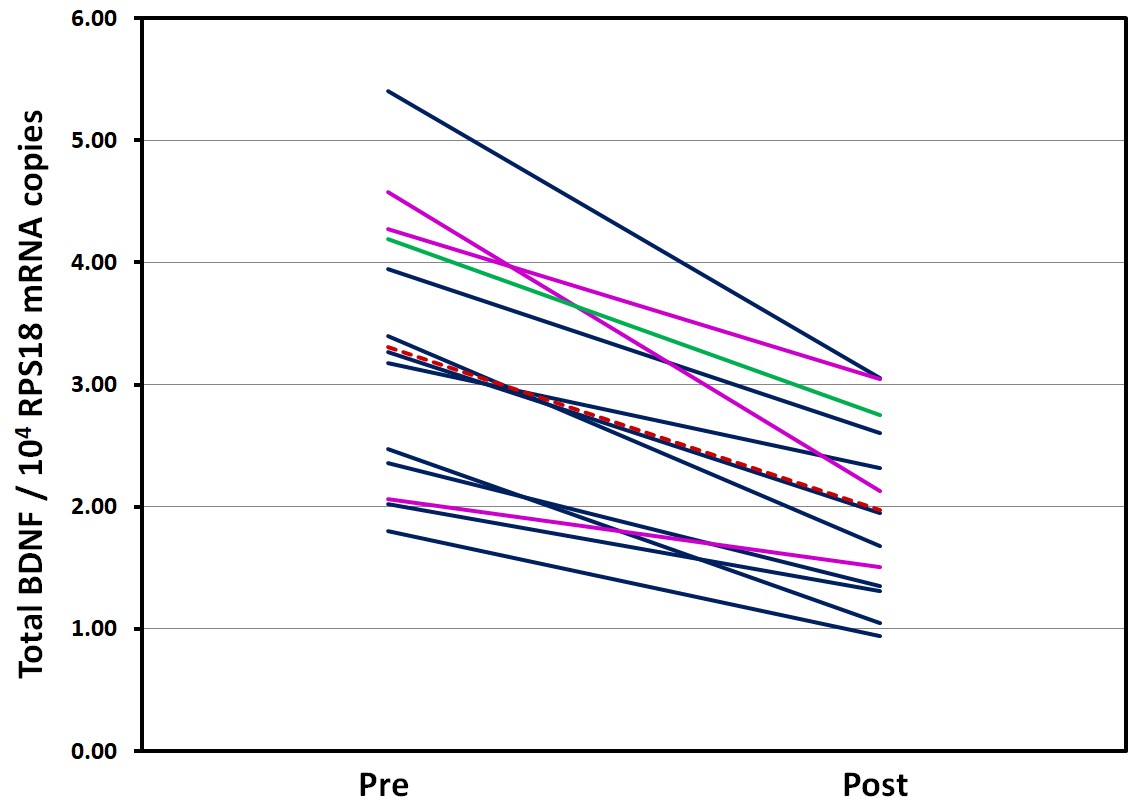

Supplement: Supplementary Figure 3 — Spaghetti plot of BDNF expression level change related to RPS18 mRNA. Pre—Total BDNF expression in rest conditions; Post—Total BDNF expression after VO2max test. Val66Val homozygotes are colored dark blue, Val66Met heterozygotes: magenta, Met66Met homozygote: green, Mean: the dotted red line. [file Image_3.jpg]
